# Biological alterations and self-reported symptoms among insecticides-exposed workers in Burkina Faso

**DOI:** 10.2478/v10102-012-0008-3

**Published:** 2012-03

**Authors:** Adama M. Toe, Sylvain Ilboudo, Moustapha Ouedraogo, Pierre I. Guissou

**Affiliations:** 1 Research Institute of Health Sciences, CNRST, Burkina Faso; 2 Laboratory of Toxicology, Health Sciences Faculty, University of Ouagadougou, Burkina Faso; 3 Laboratory of Pharmacology, Health Sciences Faculty, University of Ouagadougou, Burkina Faso

**Keywords:** occupational exposure, insecticides, hepatotoxicity, nephrotoxicity, symptoms

## Abstract

Occupationally exposed workers, farm workers and plant protection agents in the Sahel region of Burkina Faso were interviewed to assess adverse health effects of insecticides. The subjects were also examined for changes in both hematological and biochemical parameters. The prevalence of liver and kidney dysfunction was found to be quite high among insecticide applicators, especially among plant protection agents. The prevalence of biochemical alterations seems to be correlated to the frequency of insecticide use. However, no significant differences were found between the hematological parameters among farm workers and plant protection agents. The hematological parameters of all the insecticide applicators were normal. The great majority of insecticide applicators (85%) reported symptoms related to insecticide exposure. The use of insecticides in the agriculture of Burkina Faso is threatening to human health.

## Introduction

In developing countries, the losses of crops due to pests, plant diseases, and competition from weeds are great. Plant losses of the order of 40–75% have been reported, with the locust being one of the major pests responsible for losses (Clarke *et al.*, 1997). One of the strategies to increase crop productivity is effective pest management. In tropical and developing countries like Burkina Faso, crop loss is even more severe because the prevailing high temperature and humidity are highly conductive to rapid multiplication of pests (Kannan *et al.*, 1992). Thus, the application of a wide variety of insecticides on crop plants is necessary in the tropics to combat pests. Due to the role insecticides can play in the potentially increasing agricultural productivity, the Government of Burkina Faso regards these chemicals as a useful part of the agriculture. In the last few years, insecticides have extensively been applied and are still largely used by rural workers to combat locusts in the Sahelian region of Burkina Faso. However both a sporadic or a regular use of such chemicals could lead to significant consequences to public health (Abhilash and Singh, 2009; Prado-Lu, 2007). Some of the factors which are thought to have contributed to human poisoning include the lack of education and safety precautions among users and handlers of insecticides, factors which could minimize or prevent detrimental environmental and health effects (Clarke *et al.*, 1997; Ouédraogo *et al.*, 2009). Although the health hazards caused by insecticide are serious, supports from policy makers to provide for remedial measures have been lukewarm in developing countries. The resistance of policy makers is partially due to their deficient appreciation of the severity of the problem, its sources, and of suitable interventions. Their lack of knowledge is understandable, since studies of insecticide effects on health in developing countries are scarce. Moreover, neither *in vitro* toxicological approaches nor *in vivo* animal experiments are always suitable for predicting delayed adverse effects in human populations (Multinigner, 2005).

To the best of our knowledge, there is no published study about insecticide effects on exposed workers’ health in the Sahelian region of Burkina Faso, though these substances have been have used for the past two decades in agriculture. Thus, simple and effective health monitoring of those involved in insecticide application is more than essential.

The aim of this study was to assess biological signs and the prevalence of symptoms associated with insecticide exposure among insecticide applicators of the Sahelian region of Burkina Faso.

## Materials and methods

### Study area

The study was conducted in the Sahelian region of Burkina Faso from July to August, 2009. The target area is the most arid region located in the northern region of Burkina Faso. However, this area is frequently infested by locusts and/or grasshoppers, requiring the use of chemical insecticides.

### Study population

The study was cross-sectional in design. The subjects were made up of 112 insecticide applicators, comprising 84 occasional applicators (farm workers) and 28 plant protection agents (who frequently sprayed insecticides). All subjects met the criteria of not having used insecticides in the preceding three months. A convenient series was collected based on availability of the subjects on the day of the study and on their willingness to participate.

Structured questionnaires were used to collect information on socio-demographic characteristics and life work experience of the insecticide applicators. The survey also asked farmers to report symptoms experienced after mixing and spraying insecticides.

Laboratory examinations were done for each insecticide applicator. Freshly collected blood samples from the arm vein were analyzed hematologically and biochemically.

### Hematological analysis

Hematological analysis was performed using an automatic counter (ABX Diagnostics, Switzerland). Total Red Blood Cells (RBC) count (×10^6^ mm^–3^), hemoglobin content (Hb) (g.dl^–1^), hematocrit rate (HCT) (%), total number of White Blood Cells (WBC) (×10^6^ mm^–3^), total number of Platelets (×10^6^ mm^–3^), Mean Corpuscular Volume (MCV) (fl), and Mean Corpuscular Hemoglobin Concentration (MCHC) (g.dl^–1^) were assessed.

### Biochemical analysis

The serum of the blood samples was separated by centrifugation (3 000 r.p.m., for 15 min) and alanine aminotransferase (ALT), aspartate aminotransferase (AST), creatinine, and cholinesterase levels were spectrophotometrically determined using diagnostic kits (Spinreact Diagnostic Kit, Santa Coloma, Spain).

### Statistical analysis

Significant differences between mean values of farm workers and plant protection agents were statistically analyzed using Mann-Whitney's test. Chi-square (Χ^2^) or Yates corrected Chi-square test was performed to test differences between frequencies of characteristics of the study groups. Statistical analysis was carried out using GraphPad Prism version 2.01 software (GraphPad Software Inc, USA). The criterion for statistical significance was *p<*0.05.

## Results

### Socio-demographic profile

All the respondents were male. Table 1 shows the socio-demographic characteristics of the farm workers (occasional applicators) and plant protection agents.

**Table 1:** Socio-demographic characteristics (%) of farm workers (occasional applicators) and plant protection agents in the Sahelian region of Burkina Faso.

	Farm workers (n=84) No. (%)	Plants protection agents (n=28) No. (%)	Yates corrected Χ^2^ *p-value
Age (years)			
18-29	13 (15.48)	1 (3.57)	
30-49	57 (67.86)	19 (67.86)	
= 50	14 (16.66)	8 (28.57)	0.615
Mean age ± SD	39.36±9.21	41.58±10.25	

**Education**			0.014

Illiterate	55 (65.48)	6 (21.42)	
Primary school	26 (30.95)	11 (39.29)	
Secondary school	3 (3.57)	11 (39.29)	

**Training in the use of insecticides (Yes)**	69 (82.14)	28 (100)	0.002

* *p-*values are based on the distribution of the data for each characteristic, by plant protection agents and farm workers (occasional applicators)

### Insecticide exposure

In the Sahelian region of Burkina Faso, chemical insecticides used by insecticide applicators in the agricultural sector can be classified into 4 main types: organophosphates, pyrethroids, carbamates and phenylpyrazoles. The duration of insecticide use ranged from under 5 years to over 20 years. The mean duration of insecticide use was respectively 11.02±8.03 and 13.33±10.25 (*p>*0.05) in farm workers and plant protection agents. The frequency of insecticide application by plant protection agents was twice or three times a month. The farm workers used insecticides twice a year at the most. Most applications were made from June to October. Each application lasted from 30 minutes to 4 hours, and despite the high risk of exposure, farm workers did not wear proper personal protection while working with insecticides. Boots were the only protective equipment worn by the majority of farm workers (95.2%), and practically no one used aprons or gloves. Cloth face masks, which do not offer adequate coverage for some chemicals, were used by 30% of them. Improvised forms of personal protection equipment were also used, such as handkerchiefs and long sleeves. However, all the plant protection agents claimed that they used adequate personal protection equipment during insecticide application.

### Laboratory examinations

No significant differences were found between hematological parameters in farm workers and plants protection agents (Table 2). Serum biochemical parameters are shown in Table 2, while Table 3 shows frequency distribution of abnormal biochemical parameters among farm workers and plant protection workers**.**

**Table 2:** Hematological and biochemical parameters profile in farm workers and plant protection agents of the Sahelian region of Burkina Faso.

Parameters	Farm workers(n=84)Mean ± SE	Plant protection agents (n=28)Mean ± SE
Hematological parameters		
RBC (×10^6^ mm^–3^)	5.68±0.24	7.3±2.45
Hb (g.dl^–1^)	13.28±0.54	13.76±0.38
HCT (%)	45.86±2.72	45.02±1.28
WBC (×10^6^ mm^–3^)	5.11±0.16	5.16±0.15
Platelets (×10^6^ mm^–3^)	253.23±15.35	255.54±27.83
MCV (fl)	86.68±1.25	87.49±1.60
MCHC (g.dl^–1^)	30.61±0.90	30.84±0.85
Biochemical parameters		
ALT	33.44±2.45*	59.35±7.83
AST	30.67±2.57*	45.24±4.37
Creatinine (U.l^–1^)	76.57±4.12*	89.42±7.22
Cholinesterase (U.l^–1^)	2 862.34±144.10*	2 665.45±214.90

* Difference was significant (*p<*0.05) using Mann-Whitney's test

**Table 3:** Frequency of abnormal biochemical parameters among farm workers (n=84) and plant protection agents (n=28) in the Sahelian region of Burkina Faso.

Biochemical parameters	Farm workers (%)	Plant protection agents (%)
ALT	29.17*	63.16
AST	39.22*	47.62
Creatinine	19.05*	39.13

* Difference was significant (*p<*0.05) using Chi-square test

### Self-reported symptoms

**Figure 1:**
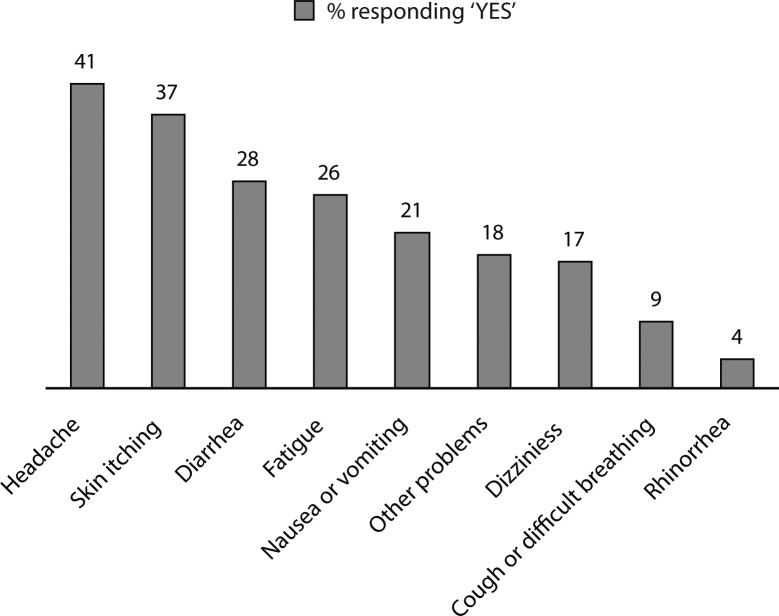
Self-reported health impairment after using insecticides (n=112).

Figure 1 displays the health complaints of insecticide applicators, possibly related to insecticide exposure. Most (85%) of the surveyed insecticide applicators reported that they had multiple symptoms after using insecticides, with an average of 3 and a maximum of 6.

## Discussion

Some practices predisposing to insecticide exposure and illness were identified in insecticide applicators in the Sahelian region of Burkina Faso. The mean duration of insecticide use was more than ten years (about 11 and 13 years for farm workers and plant protection agents, respectively). This is very significant, and indicates chronic exposure among these applicators. Most insecticide applicators were illiterate (Table 1) and did not wear proper personal protection. This shows the seriousness of the situation faced by the insecticide applicators. In Poland, 22 poisoning cases were seen in 2002 as a result of spraying pesticides without adequate protective equipment (Przybylska, 2004).

All the hematologic parameters of the two study groups (farm workers and plant protection agents) were normal. Similar results were reported by other authors (Lebailly *et al.*, 2003; Pastor *et al.*, 2002). However, many hematologic changes secondary to acute and chronic insecticide exposure were reported in both humans and animals, although there are some conflicting results (Jamil *et al.*, 2007; Meaklim *et al.*, 2003; Saly *et al.*, 1995). Insecticides were shown to have hematotoxic properties and may cause aplastic anemia, agranulocytosis, neutropenia, and thrombopenia (Parent-Massin & Thouvenot, 1993). The contradictions may be attributed to the types of insecticides used and to exposure conditions.

The increase in the level of ALT and /or AST is a good indicator of hepatic toxicity (Dorosz, 2002; Hall, 2001). The mean levels of ALT and AST in plant protection agents were higher than the levels in farm workers (Table 2). Liver dysfunction (abnormal rise of ALT and AST) was most frequent (63.16% and 47.62%, respectively) among plant protection agents (Table 3). Abnormal levels of transaminases (ALT and AST) were found also in occupationally insecticide-exposed workers in India and Pakistan. The changes were related to the exposure of workers to insecticides (Azmi *et al.*, 2006; Hernandez *et al.*, 2006; Khan *et al.*, 2008; Patil *et al.*, 2003).

The mean level of creatinine concentration in plant protection agents was also higher than the level in farm workers (Table 2). The prevalence of abnormal levels of creatinine among plants protection agents (39.13%) was higher than the prevalence in farm workers (19.05%) (Table 3). A previous study reported subtle nephrotoxic changes in workers occupationally exposed to insecticides with high levels of creatinine (Attia, 2006).

Although organophosphate insecticides were also used by insecticide applicators, serum cholinesterase level was normal (between 1 900 and 3 800 U.l^–1^) (Dorosz, 2002) in all farm workers and plant protection agents. This is not surprising because serum cholinesterase indicates a significant acute organophosphate toxicity (Prado-Lu, 2007). However, the mean serum cholinesterase level of plant protection agents was lower than that of farm workers (Table 2). A screening of Hmong farmers in Thailand who used insecticides in their fields showed that 20–69% of 582 Hmong adults had risky or unsafe levels of cholinesterase (Kunstadter *et al.*, 2001).Cholinesterase corresponds to two enzymes: acetylcholinesterase and butyrylcholinesterase. The latter is also called plasma cholinesterase (Hernandez *et al.*, 2004). The activity of cholinesterase enzymes in blood can be utilized as a biomarker for the effect of organophosphates. An intoxicated person will show abnormally low levels of activity of cholinesterase enzymes measured in serum or in red blood cells (Kachaiyaphum *et al.*, 2010; Tinoco-Ojanguren & Halperin, 1998).

The present study revealed that the prevalence of liver and kidney dysfunction was high among insecticide applicators in the Sahelian region of Burkina Faso and this prevalence was higher among plant protection agents. This situation could be attributed to inappropriate protection equipment of the applicators and to the frequent use of insecticides by plant protection agents.

In our study, headache was the most frequently reported symptom (40.61%), closely followed by skin itching (37%), diarrhea (28%), fatigue (26%), and nausea or vomiting (21%). Dizziness, cough or difficult breathing and rhinorrhea were also common (respectively 17%, 9%, and 4%). Such a constellation of symptoms is consistent with previous findings among farmers exposed to organophosphate insecticides (Strong *et al.*, 2004). Our results highlight the high frequency of symptoms among insecticide applicators (85%) and confirm that they did not wear proper personal equipment while working. In northern Vietnam, Murphy *et al.* reported that, out of 1798 recorded pesticides spray operations 8% of the subjects involved were asymptomatic, 61% presented with vague ill-defined effects, and 31% were affected with at least one clear symptom of poisoning (Murphy *et al.*, 2002).

The design of our survey did not allow to report on serious and long-term consequences of insecticides. Some authors observed that exposure to insecticides was increasingly linked to immune suppression, hormone disruption, diminished intelligence, reproductive abnormalities, and cancer (Undeger and Basaran, 2005; Varona *et al.*, 2003).

## Conclusion

The study demonstrated the detrimental effect of exposure to insecticides on hematologic and biochemical parameters of insecticide applicators in the Sahelian region of Burkina Faso. The laboratory examinations revealed that the prevalence of liver or kidney dysfunctions was quite high among insecticide applicators, especially among plant protection agents. The prevalence of biochemical alterations seems to be correlated to the frequent use of insecticides. No significant differences were found between hematological parameters in farm workers and plant protection agents. The great majority of insecticide applicators reported symptoms associated with insecticide exposure. More detailed studies investigating other delayed effects of insecticides on human health are critically needed in Burkina Faso. Intensive intervention efforts to reduce insecticide mortality and morbidity are required.

## Acknowledgments

The authors are grateful to DPV (Direction de la Protection des Végétaux) and PULCPA (Projet d'Urgence de Lutte Contre le Criquet Pèlerin en Afrique) for their financial and technical assistance.
